# Striving towards integrated people-centred health services: reflections on the Australian experience

**DOI:** 10.1136/fmch-2018-000056

**Published:** 2019-01-29

**Authors:** David G Legge

**Affiliations:** La Trobe China Health Program, La Trobe University, Bundoora, Victoria, Australia

**Keywords:** delivery of health care, integrated, health policy, public health systems research

## Abstract

Health systems around the world are under continuing pressure for reform. Health system reform involves both content and process. Content deals with changes to the structures of the health system; process deals with the strategies of change. In this paper, we reflect on the development of the Australian healthcare system and draw out lessons regarding both structural and developmental principles. We review the historical development and functional performance of a range of ‘programmes’ which comprise the Australian health system. We use WHO’s 2016 ‘framework on integrated people‐centred health services’ as a standard against which to evaluate the performance of the different programmes. A model of health system development featuring incremental change, windows of opportunity and policy coherence is used to frame some lessons from the Australian experience regarding reform strategy. Several of the programmes reviewed can be shown to have contributed positively to integrated and people-centred services. However, there have also been significant shortfalls in performance. The successes and the shortfalls of the programmes reviewed reflect both their histories and their contemporary context. Structural principles emerging from this review include the policy leverage available under single payer purchasing and on the other hand the fragmenting effects of privatisation and marketisation. Lessons regarding strategies of reform include cultivating ‘reform readiness’ across all of the locations and levels where opportunities for change may emerge while cultivating system wide coherence through a shared vision of how the system as a whole should develop.

## Integrated people-centred health services

At the core of the idea of ‘integrated people‐centred health services’^1^ are three aspects:

- patient‐centred healthcare,
- community‐centred health services and
- improved coordination across providers and sectors.

Patient‐centred healthcare is a critical element of the framework. It includes: the integration of different services around the needs and wants of patients; care which understands and responds to family and social context and respectful, participatory, accountable service delivery. Patient-centred care is not centred on provider interests, body parts, particular diseases or particular technologies.

Integrated people-centred health services go beyond the individual patient to encompass the community: community‐centred programme delivery and community centred health planning. Community-centred programme delivery is respectful, responsive, participatory and accountable. Community-centred planning, including facility development, workforce development, programme planning, is likewise respectful, responsive, participatory and accountable.

Improved coordination is a critical part of integrated and people-centred services. This includes coordination: of the care provided to individuals; of services from different providers and coordination across different health programmes. It also includes coordination across levels of administration; across the public and private sectors and across health and non-health sectors of administration.

## Integrated people-centred health services: a vision

Integrated people‐centred health services includes patient‐centred care, community‐ centred service delivery and planning and improved coordination across providers and sectors.

However, integrated people‐centred health services is not a policy framework; it is not a model of service delivery; it is not a pathway for service development. It is a vision; a vision which, if widely shared, can provide coherence across the many different decisions, taken at different times in different bureaucracies and at different levels, which, in aggregate, shape the delivery of health services and programmes and the development of health systems.

It is a vision which has the capacity to take us beyond a number of dead ends in global health policy development:

- beyond the narrow, vertical, disease‐oriented programmes^2 3^; in particular beyond the silos established to manage development assistance for health, which contribute to the fragmentation of programmes, high transaction costs and internal brain drain;
- beyond universal health coverage which in essence is just about healthcare financing; health policy must be understood as more than simply paying for healthcare;^4 5^
- beyond slogans about the social determinants of health to actually integrating practical action on the social determinants of health into mainstream health services;^6^
- beyond incentive engineering with incentives tied to more and finer performance indicators which compromise a more holistic view of the ‘patient journey’ and relationships of trust;^7^
- beyond marketisation policies through which healthcare is reduced to the buying and selling of commodities which also compromises the holistic perspective and relationships of trust;^8^
- beyond privatisation, which, in the presence of wide income inequality, leads to stratified services (from VIP care to base level safety nets) which further weakens social cohesion and social solidarity.^9 10^

It is a vision beyond the blockages.

## The Australian experience

In this section, we reflect on the Australian experience of health system development against the principles of integrated people‐centred health services.

We describe some interesting models from Australia which have contributed positively towards integrated people‐centred services and reflect on the histories of those models, looking for more generally applicable lessons in terms of health systems development.

We also describe some aspects of the Australian health system which negatively impact on integration and people‐centredness and we will reflect on causes of such weaknesses and possible lessons in terms of health system planning.

### Medicare: universal single payer health insurance

Medicare is Australia’s universal single payer health insurance system.^11^ It provides universal coverage (covering most of the cost of most services for almost all people).

The fact that it is a single payer system provides important policy levers over quality and efficiency. Because it is largely funded from taxation, it incorporates a significant redistributive function from wealthy to poor as well as protecting against the risk of ill‐health.

The key to the introduction and stability of Medicare has been popular support. It was originally introduced with strong support from the trade union movement and has proved to be a very sensitive issue at election time; politicians who threaten to dismantle Medicare lose votes.

Some of the lessons we draw from the Medicare example are the importance of an informed and engaged public constituency, a clever policy narrative for that constituency to sign up to and political leadership.

However, Medicare is not perfect; conflict between the Federal and state governments is common, largely over funding, including frequent attempts at cost‐shifting where programme planning is directed to shifting the cost burden to the other tier of government. Policy control over public spending through the private sector is quite weak.

### The Pharmaceutical Benefits Scheme (PBS)

The PBS is the national drugs purchaser with a number of significant benefits flowing from its role as a monopoly purchaser.^11^ The prices which are paid to manufacturers are based on rigorous cost effectiveness analysis with a premium paid for more effective drugs (while ‘me-too’ drugs are discounted). Monopoly purchasing provides significant policy leverage in terms of utilisation review, remote supply, rationing of expensive drugs and shortage control.

The establishment of the PBS reflects years of policy pressure through the Australian Labor Party, often over fierce opposition from private medicine and the Liberal Party.^12^ Over the years, the PBS has attracted increasing public support as well as political support from the medical profession and the pharmaceutical industry. It is now firmly entrenched.

In terms of policy process the key lessons are about public support, clever policy and political leadership.

However, the PBS is not without its weaknesses. There is weak control over excessive and inappropriate prescribing and very weak control over pharmaceutical marketing which has a major effect on price setting and prescribing.

### National Health Funding Pool and local hospital networks

The National Health Funding Pool (NHFP) and the local hospital networks are two recent initiatives which are directed to improved coordination between Federal and state governments, between hospitals and between hospitals and community health services.^13^

Some states had been using diagnosis related groups (DRG)-based funding of acute inpatient care since the early 1990s but in 2013, when the National Funding Pool was established, DRG funding (so-called ‘activity based funding’) was standardised across the whole country.

DRG funding of inpatient care promotes efficiency in the delivery of the episode of care (assuming the propensity for gaming is controlled). It provides managerial discretion for facility managers; and it provides high quality information and strong policy levers for the system managers.

The NHFP notionally pools funding for hospitals from both the Federal and state levels in a relatively transparent arrangement which is designed to control cost shifting between the two levels of government. Cost‐shifting, where policy is directed to shifting the cost burden to the other tier of government, has been a blight on health system development in Australia for many years.

With the NHFP came the establishment of ‘local hospital networks’ which bring together hospitals serving particular regions.^14^ Part of the objective was to enable the Federal government to have a direct relationship with local hospital network boards, hitherto purely creatures of state governments, in order to promote transparency and standardised data systems.

The LHNs are also expected to improve the coordination of facility development and programme planning across the hospital networks and to facilitate improved coordination between hospitals and community health services through the establishment of a parallel system of ‘primary health networks’.

Possible lessons from this story are about clever policy and bureaucratic leadership. Public awareness of these structures is low and likely to remain so. While these structures address a critical domain of health policy (federal state relations), they are not strongly driven by public sentiment.

Significant challenges remain. DRG funding of hospitals carries its own perverse incentives which need to be managed. The incentives for cost shifting remain strong and the attempt to apply the principle of ‘activity based funding’ across the whole system is doomed.

### Quality and safety: guidelines, measurement, accountability

Australia is making slow progress in relation to quality and safety with increasing investment in evidence based clinical guidelines, standardised measurement of both process and outcome indicators and stronger accountability through formal structures of clinical governance.^15^

Progress in the adoption of evidence‐based medicine has contributed to firmer clinical guidelines. Support for innovation and evaluation in quality improvement and patient safety has contributed to improved technical understanding of the models, metrics and culture of clinical governance. The cost argument for greater clinical accountability has assumed greater salience with increased public expenditure and better information to the public (particularly through the media) has given a political edge to the debate.

Some of the possible lessons which might be drawn from this experience involve:

- the importance of information development (and publication); the quality, timeliness and accessibility of meaningful clinical information;
- the powerful impact that scandals and media exposés can have on electoral sentiment and
- the importance of clever policy, including support for innovation and evaluation.

However, not all is perfect. Progress so far has largely focused on acute inpatient care. It has not been so strong in private medicine, private hospitals, aged care, disability care or community care.

### Community health (general practice, community nursing, pharmacy and allied health)

The local network of community health services, including general practice, community nursing, community pharmacy and allied health, is a foundational asset of the Australian health system.^11^

These generalist primary healthcare practitioners operate in every district and generally work fairly well together. They carry high levels of community trust. It is a system with a very long history, built on private general practice and small business pharmacy.

With increasing public funding there has been a corresponding increase in policy attention to developing this system, including better coordination and stronger accountability.

There are some possible lessons which are worth considering. One is the degree to which health system development builds on the institutional and cultural legacies from the past. This points to the need for creative policy making (rather than copying) because the history of each system is different. Transforming the legacies of the past into the institutions, we need for tomorrow calls for a high level of policy capacity. Another possible lesson is about the importance of building the constituencies for change (professional, institutional, bureaucratic and community constituencies). Changing long established institutions and cultures is always politically sensitive and without constituencies for change it would not happen.

There remain significant weaknesses. There remain continuing barriers between public and private services in community health and between programmes funded by different sectors or different levels of government. There remains a huge challenge to strengthen the role of community health services in prevention, including action on the social determinants of health.

### Two way referral relations between GP and specialists/hospitals

A key feature of the Australian system is the two way referral and report relationship between GPs and medical specialists including in public and private hospitals.

The GP is not just a ‘gate‐keeper’; she is also a broker, helping patients to find their ways through the specialist system. At its best, this involves direct communication between the GP and specialist when referring patients and a detailed report back to the GP from the specialist at appropriate intervals during the patient’s care.

In recent years, there has been an increasing policy focus on the role of the GP in preventing avoidable hospital admissions and in postdischarge care. This involves closer coordination across these levels but it tends to be driven by the pressure on hospital beds rather than the optimal patient journey.

Of course the system does not always work as it is supposed to. In many cases, GPs exercise their brokering function without systematic information about the performance level of the specialists they are recommending. Reports from public hospitals to GPs vary in quality and timeliness.

The GP brokerage/gate‐keeping role has a long history. It emerged originally in the great compromise of 1858 in the UK when three different streams of medical practice were brought together in one profession. It has been reinforced in Australia through paying a higher level of specialist medical insurance benefits for services provided on referral from the GP. The lessons are as before: the health system develops on the legacies from the past and this demands clever policy, political leadership and constituency building.

### Primary health networks

General practice in Australia started out as a privately owned small business, based on solo practice and fee for service remuneration. These structural origins gave rise to a culture which was highly individualistic and fiercely defensive. But this is changing. Group practice is now the norm and full or part time salaried appointments in general practice are becoming more common.

As public funding for healthcare increases, there is an increased policy focus on the cost-effectiveness of public expenditure. One manifestation of this has been continuing efforts to create regional bodies, based on general practice, to plan for and coordinate service delivery in the PHC sector and to promote coordination between primary and tertiary sectors.^16^

These policy initiatives have moved from being largely consultative and GP‐focused to including a wider range of stakeholders and a broader remit including ‘commissioning’ of new services. This is a work in progress. Promoting improved coordination still relies largely on financial incentives and GP is a long way from understanding let alone addressing the social determinants of population health.

The lessons, yet again, centre on the challenges of achieving incremental change, building on the legacies of the past. There is no public constituency for or against primary health networks. The debate has been almost entirely between the policy logic of the technocrats and the resistance of medical practitioners to what they perceive as the whittling away of their professional privileges. Political leadership from government and from within the profession has proved critical.

### Dental services

There are several sectors where integrated and people-centred services are still a way off in Australia and in this and the following sections, we explore some of the barriers to progress in this direction.

There are wide inequalities in dental health status in Australia and dental services are split between an expensive private sector with high out of pocket costs and very limited insurance cover and a weak public sector, restricted to children and emergencies and with long delays for treatment.^17 18^

There are two main barriers to integrated people‐centred dental healthcare in Australia. One is the political power of the private dental profession and its long standing opposition to extending salaried dental services. The second is the reluctance of government to include dental cover under Medicare because of the difficulty in controlling volume and costs in a fee for service system.

Possible lessons from this experience are about the need for political leadership, clever policy and constituency building to manage the transition to a more equitable, accountable and efficient system. Universal and equitable access to dental care depends in part on the strength of social solidarity which is not easy to maintain in the face of market fundamentalism and widening economic inequality.

### Mental health services

In mental health, there is a wide gap between services for anxiety and mild to moderate depression, which are largely provided by Medicare‐covered GPs, psychiatrists and clinical psychologists as compared with services for people with ‘serious mental illness’, which are provided largely through public facilities.^19^

Private Medicare funded services attract generous insurance benefits but there are only loose controls on volume and none on quality/effectiveness. In contrast, public services, in acute inpatient units and through community mental health teams, are seriously underfunded. Likewise, funding support for public housing and supported accommodation is inadequate. Many people with chronic mental illness have quite unstable living arrangements and there is a widespread problem of homelessness among clients of the state system.

The barriers to integrated and people‐centred mental health services in Australia can be traced historically to the early development of government mental health services and the subsequent separate and parallel emergence of private psychiatry, latterly supported through medical insurance.

Public mental health services went through a revolution in the 1950s and 1960s with the emergence of drug treatment of schizophrenia and other illnesses. An apparently progressive policy thrust to ‘deinstitutionalisation’ (and the sale of the old style mental health facilities) during the 1970s proved to be a bonanza for state treasury (from property sales) but too little was invested in modern programmes of care, including supported accommodation.

Looming behind these policy failures is the rising tide of neoliberalism, including the retreat from the welfare state, with lower safety nets and more general acceptance of widening economic inequality.

One possible lesson from this experience is that navigating the transition from the legacies of the past is not always successful, particularly at a time of weakening social solidarity and widening economic inequality.

### Private health insurance and private hospital services

Another barrier to integrated and people‐centred health services in Australia is the lack of accountability of private hospitals, private specialists and private insurance funds for the quality of private hospital care and the efficiency with which public funds are used. Conservative governments have legislated for huge government subsidies to keep the price of private health insurance (for private inpatient care) affordable while public hospitals are seriously stretched. Significant financial penalties (linked to Medicare premiums) have been put in place to encourage (or force) people to buy private hospital insurance.^11^

This gulf between the public and private hospital sectors reflects the political power of private medicine, the private hospital owners and the private health insurance companies. It also reflects public concern about delays in the public sector and a preference among middle-income and higher-income families for private hospital care and ‘choosing one’s own specialist’ (which is not routine in the public hospital sector).

There are possible lessons here about the capacity of powerful vested interests to preserve their privileges notwithstanding the technocratic logic of policy reform. Stalled policy development in this case also reflects different perspectives within the wider community about the role of private hospital care.

This experience underlines the need for information and transparency, for clever policy, for political leadership and for constituency building for implementation.

### Aged care

Residential aged care in Australia is heavily subsidised by the federal government but largely delivered by private providers. The government seeks to maintain tight control on aged care benefits which leads providers to support continued profit-making by skimping on staff and quality of services. However, the government depends on the private providers staying in the field, despite inadequate funding, so it turns a blind eye to shortfalls in quality and safety until forced by public opinion to increase the subsidy.

The barriers to integrated and people‐centred health services in the aged care sector reflect political forces, policy models and societal trends, many of which arise well beyond the health and aged care sectors.

## Conclusion

This brief review, of Australian progress towards integrated and people‐centred health services, points towards two sets of broad conclusions regarding structural and developmental principles.

### Structural principles

Some structural principles which emerge from this review include:

- single payer purchasing (evidence‐based, transparent and accountable) strengthens the policy levers needed to achieve public goods outcomes across the system;
- incremental adjustments to funding modalities can help to align more closely the financial incentives the and the policy objectives;
- nudging provider performance involves honouring professionalism and strengthening accountability as well as incentive engineering;
- investment in innovation and evaluation informs clever policy and constituency building;
- investment in meaningful measurement contributes to constituency building and accountability;
- addressing the barriers to cooperation across providers, across levels of government and between different sectors of administration requires structural reform, institutional culture change and strengthened public accountability (including media pressure).

The prevailing neoliberal policy framework constitutes a major barrier to people-centred health services:^20^

- privatisation and marketisation fragment service delivery and weaken policy control;
- equitable universal access is not consistent with discriminatory safety nets;
- global pressures for lower taxation are contributing to a widespread and regrettable retreat from the welfare state.

### Developmental principles

The ‘lessons’ regarding the processes of health system development, which we have drawn from our review of the Australian experience, may be systematised in accordance with Kingdon’s ‘three streams’ model of policy implementation.^21^

Health systems develop through incremental change. Such change takes place when established institutions unfreeze (often because of increasingly evident dysfunction), *and* there are clever policies on hand, *and* public interest constituencies are driving towards a coherent vision for change.

Opportunities for change arise unpredictably; both in terms of timing and the institutional location of such unfreezing. Accordingly, the system as a whole, and all of the various loci of possible reform, need to be prepared so that opportunities can be converted into the implementation of progressive change when they emerge. Creating such ‘reform readiness’, locally and generally, involves:

- building consensus around why change is necessary;
- building the policy dialogue among stakeholders, including various affected communities, to develop clever policies for different contingencies;
- strengthening the political leadership needed to drive change.

Building a shared vision of how the system as a whole should be developing is a critical part of this model to ensure that incremental changes taking place at different times and places are coherent and synergistic. WHO’s framework on integrated and people-centred health services provides a vision of the kind of health system we are striving towards.

Promoting this vision, engaging stakeholders in policy dialogue and supporting the emergence of community constituencies whose interests will be served by such policy reform are critical tasks for policy leaders.
